# Designing a mentorship programme for women football coaches in South Africa: an expert e-Delphi study

**DOI:** 10.3389/fspor.2026.1821565

**Published:** 2026-07-06

**Authors:** Thembisile Mbatha, Heather Morris-Eyton

**Affiliations:** Department of Sport and Movement Studies, Faculty of Health Sciences, University of Johannesburg, Johannesburg, South Africa

**Keywords:** coaching, e-Delphi, football, mentorship, women coaches

## Abstract

**Introduction:**

Despite global recognition of mentorship as a key strategy for advancing women in sport coaching, structured mentorship pathways for women football coaches remain limited within the South African context. While national sport policies emphasise equity and transformation, there is little empirical guidance informing the design of contextually relevant mentorship programmes. This study aimed to establish expert consensus to inform the development of a mentorship programme tailored to women football coaches in South Africa.

**Methods:**

An expert e-Delphi methodology was employed to systematically gather and refine perspectives from a panel of football experts with experience in coaching, coach education, and football administration. Thirteen experts participated in the initial round, with twelve completing the subsequent consensus rounds. Initial rounds generated statements relating to mentorship structure, objectives, delivery mechanisms, and support systems, which were refined through iterative feedback and consensus-building processes.

**Results:**

The findings identified key elements necessary for an effective mentorship programme, including structured matching processes for mentors and mentees, clear role definitions, capacity-building components, organisational support, and monitoring mechanisms. Consensus was achieved on the core components of the mentorship programme, resulting in the development of a proposed mentorship framework. Experts emphasised the importance of embedding mentorship within formal coaching pathways rather than relying solely on informal relationships.

**Discussion:**

This study provides evidence-based, context-specific guidance for the development of structured mentorship initiatives aimed at advancing women football coaches in South Africa. The findings highlight the value of formalised mentorship structures in supporting coach development and contribute to broader efforts to establish equitable, sustainable, and inclusive coach development systems within South African football.

## 1 Introduction

Although women's participation in football has increased substantially worldwide, representation in coaching and leadership positions remains disproportionately low (1–4). Historically, coaching structures have been defined by male dominated norms and practices, that restrict women's access to career progression, and leadership pathways. In the African context, these challenges compounded by broader structural and organisational constraints within the football system, resulting in limited and inequitable development opportunities for women coaches (5). Consequently, mentorship has emerged as a strategy to support career progression and address persistent disparities in sport coaching.

Globally, the sport coaching literature recognises mentorship as a mechanism for developing and retaining underrepresented groups in coaching (6–8). In football, mentorship facilitates experiential learning, builds confidence, and provides career guidance, traditionally more accessible to men. This has led to international governing bodies integrating mentorship into broader coach development and gender equity strategies (2, 9–11). South African initiatives remain largely informal, inconsistent, or absent. Where mentorship does exists, it is often reliant on individual relationships, rather than being embedded in formal organisational oaching pathway programmes, limiting its long-term impact and sustainability.

Women football coaches in Africa navigate universal barriers identified in the sport coaching literature. These include but are not limited to a lack of financial and infrastructural resources, inconsistent coach education opportunities, male-dominated football culture or environment, and limited organisational support structures (12). These are often intensified by contextual constraints or challenges (13). Where informal support networks and individual mentoring relationships occur, there is aa distinct lack of formalised mentorship initiatives designed to support women coaches across the African football system. Consequently, research focusing on mentorship as a developmental mechanism remains scarce, revealing a significant gap in the evidence required to design contextually relevant and sustainable programmes. While mentorship is widely recommended to mitigate gendered barriers, there is limited evidence examining how these programmes are designed, implemented, and evaluated, particularly within the African context. Moreover, the perspectives of football experts, including coach educators, administrators, and experienced practitioners, remain underrepresented in the literature, despite their critical role in shaping coaching pathways and development structures.

While women's football has seen significant growth in participation, international recognition, as well as competitive performance (14, 15), the representation of women in coaching continues to reflect persistent gender disparities. In South Africa women coaches must navigate intersectional challenges related to gender, race, access to qualifications, and professional networks within a historically male-dominated football system (2, 13). Although national sport policies, including the National Sport and Recreation Plan (May, 2012) and the Department of Sport, Arts and Culture's (July, 2024), addressed through the Women in Sport framework, prioritise equity and inclusion, there is limited evidence that these commitments have translated into structured, sustainable mentorship programmes. This disconnect between policy intent and practical implementation underscores the need for evidence-informed mentorship frameworks tailored to the South African football context.

Internationally, governing bodies such as the Fédération Internationale de Football Association (FIFA), and the Union of European Football Associations (UEFA) have recognised mentorship as a strategic tool for advancing women in football leadership and coaching. FIFA's Women's Development Programmes and UEFA's Women in Football Leadership initiatives include structured mentorship components aimed at supporting women's progression into coaching, technical, and leadership roles (16). While these programmes provide models for formalised mentorship, their design and implementation are largely shaped by Global North contexts. As such, their direct transferability to African and South African football environments remains limited without contextual adaptation informed by local expertise.

Given the discrepancy between global models and regional complexities, it is essential to engage local football experts to design programmes that are feasible, contextually relevant, and responsive to the lived realities of South African women football coaches. The e-Delphi method provides a rigorous, consensus-based approach for capturing expert perspectives, particularly in practice-oriented fields where empirical research is scarce (17). Through iterative feedback and refinement, this methodology identifies key mentorship components, structures, and priorities for effective programme development.

Accordingly, this study utilises an e-Delphi approach to synthesise football expert insights and establish a consensus-based framework for mentorship. Figure 4 presents the proposed mentorship programme developed through this consensus-building process. By establishing expert consensus, this study provides evidence-based guidance for mentorship design that aligns with global practice while remaining fundamentally sensitive to the specificities of the South African football landscape.

## 2 Methods

### 2.1 Research design

The Delphi method is a systematic qualitative method of interactive forecasting by collecting opinions from a group of experts through several rounds of questions to achieve consensus on a specific topic (48). Where there is a lack of evidence, the Delphi provides an opportunity to gather information or opinions from experts from different settings and geographical locations. A qualitative, consensus-based e-Delphi method (using an online platform) was used to gather, explore, and refine experts' opinions regarding the key components required for the development of a mentorship programme for women football coaches in South Africa. It enabled the systematic collection and refinement of experts' knowledge through an iterative, confidential process that reduces individual bias and promotes collective consensus.

### 2.2 Recruitment and participants

**Figure 1:**
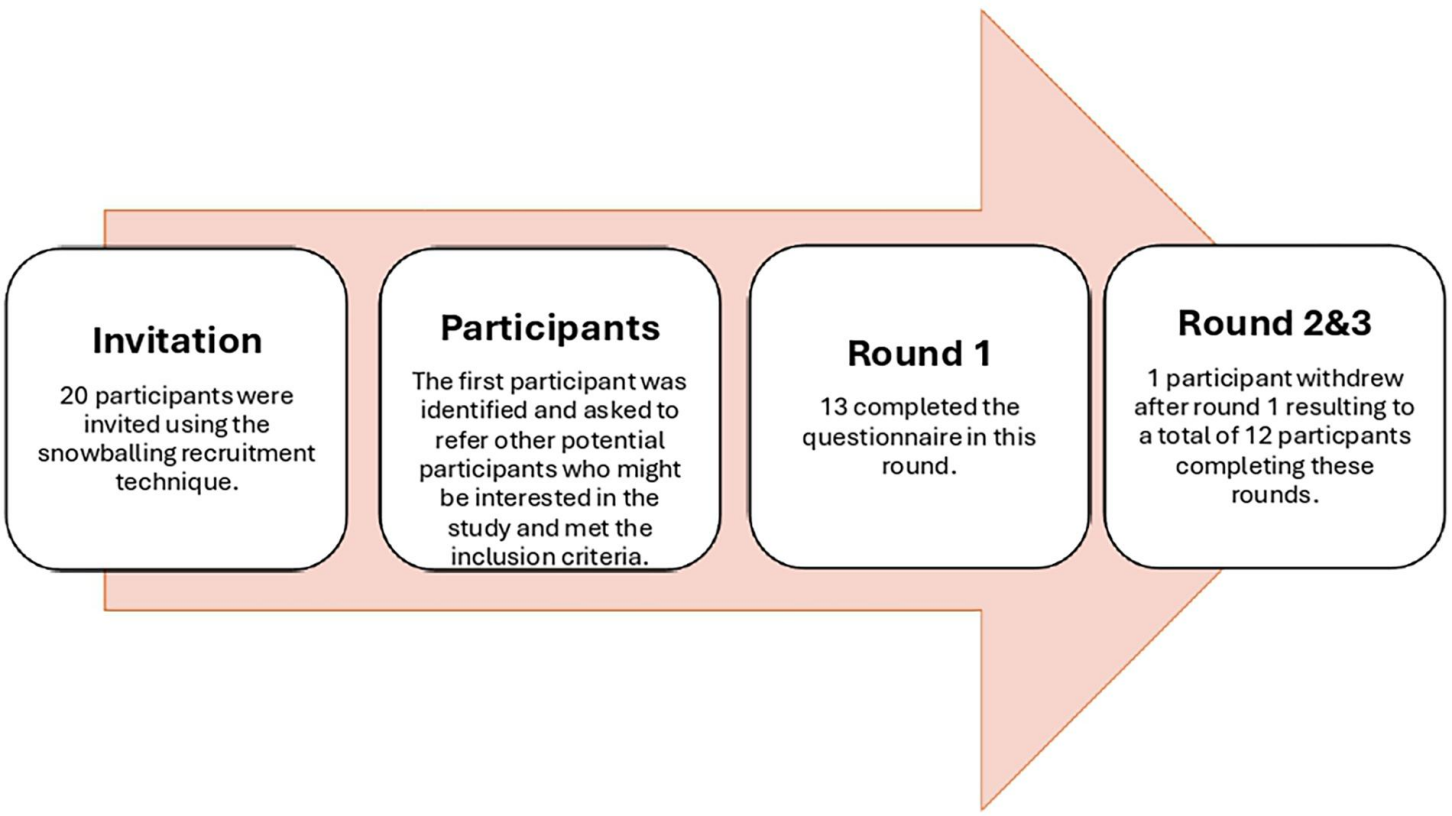
Recruitment process.

Experts are recognised as professionals who are valued for their rich knowledge and often sought out for advice, direction, or solutions (49). The experts identified for inclusion in this study were male and female mentors who came from various provinces in South Africa as well as internationally, and who hold a SAFA B or above licensing certificate, with a minimum of five years' experience in football coaching, sport administration, coach education, or mentorship in football. A snowball technique was used to recruit and invite experts to participate in the study. The initial participants were encouraged to refer other potential experts who might be interested in the research or who could provide rich knowledge (50). Thirteen experts were initially recruited with twelve completing the full process (Figure 1), which was deemed sufficient for consensus-building in specialised fields (18).

### 2.3 Procedure

**Figure 2:**
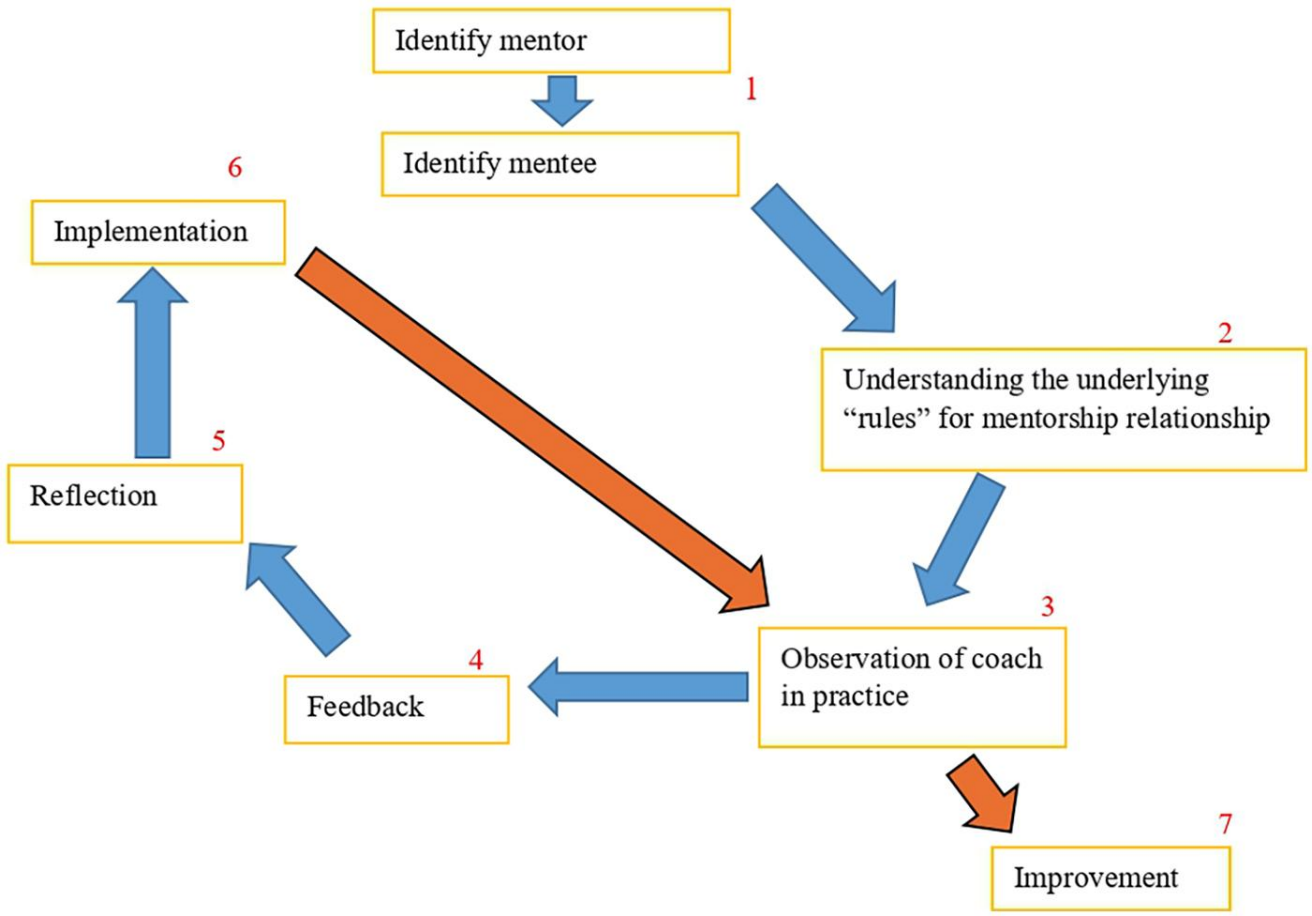
Adapted mentorship cycle from the coach mentor programme developed by New Zealand cricket (2010).

**Round 1** involved the distribution of an open-ended e-Delphi questionnaire to the panel of experts (*n* = 13), following an initial screening based on the inclusion criteria. Prior to this, the questionnaire was piloted with a small group of participants who were excluded from the main study. Experts received a Google Forms link, along with an explanation of the study purpose and procedures via email, and all participants provided electronic consent (REC-2627-2024). The open-ended responses were analysed using thematic analysis, where the resulting themes informed the development of structured statements for **Round 2**. In this round (*n* = 12 due to a withdrawal of a participant), statements derived from the round 1 coding process were presented to all experts, who rated each item using a five-point Likert scale (1–5) and were able to provide comments or suggest modifications where disagreement occurred. In **Round 3** experts were provided with synthesised sub-themes, definitions, descriptions, and a mentorship programme cycle (Figure 2). This approach enabled informed judgement without additional iterative rounds. The final statements were then circulated electronically for programme content validation, with experts indicating approval or disapproval of each statement.

### 2.4 Data analysis

**Figure 3:**
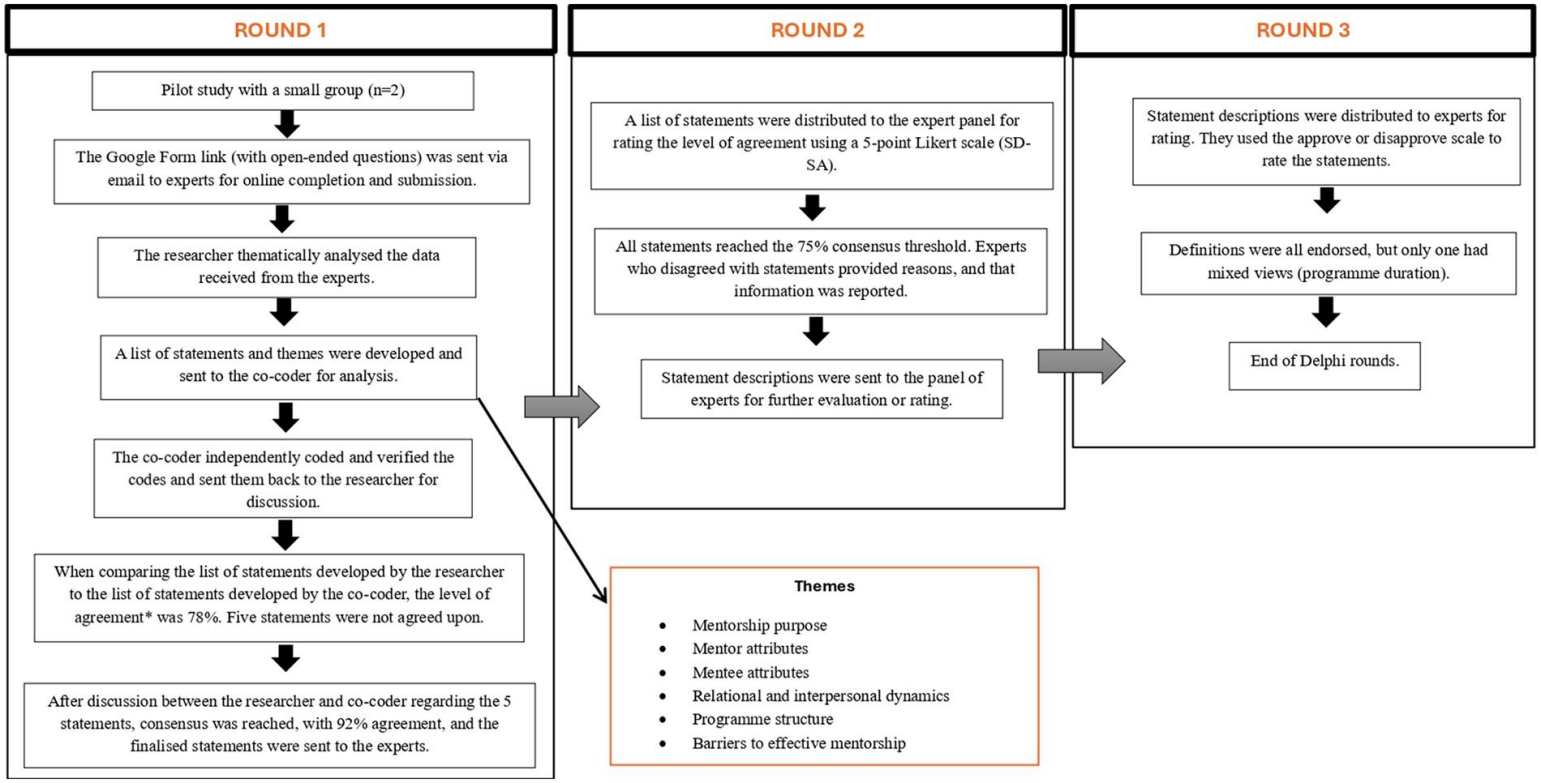
Delphi process (round 1–3).

The data from round 1 were analysed using thematic analysis. These themes informed the development of structured statements for round 2. In round 2, participants rated each statement using the five-point Likert scale to indicate the degree of agreement. In the final round, participants were asked to indicate their final judgments on each statement using a binary approve or disapprove scale. The proportion of experts who approved each statement was calculated to assess consensus. Consensus was reached if the agreement percentage was ≥75% (Figure 3).

### 2.5 Reliability and validity

Round 1 questionnaire was piloted with individuals meeting the inclusion criteria but excluded from the main study, strengthening clarity. Content validity was reinforced through the iterative three-round design, with thematic analysis of round 1 responses informing the development of structured statements for round 2.

Reliability was supported by a structured rating process using a five-point Likert scale, consistent application of consensus criteria, and opportunities for qualitative feedback. In round 3, the process was adapted to minimise attrition, with experts reviewing content that will be consolidated into a mentorship framework.

### 2.6 Data management and ethical compliance

Data was collected using Google Forms, and participant recruitment was conducted via email. All responses were anonymised prior to analysis, with no personally identifiable information included in the dataset. Data was securely stored on password-protected devices and institutional servers, with access restricted to the primary researcher and the supervisor. In line with institutional policy, data will be retained for a period of five years and thereafter securely deleted.

**Table 1:** Level of agreement between the coders in round 1.

**Questions**	**Round 1**	**Round 2**
How would you define the term mentorship?	74%	80%
What qualities and experiences should **mentors** possess to effectively support and guide female mentees in the football industry?	87%	91%
What do you think the roles and responsibilities are for a **mentor** in a mentorship programme?	68%	94%
What do you think the roles and responsibilities are for a **mentee** in a mentorship programme?	60%	91%
What criteria do you recommend for selecting **mentors** in a programme aimed at female coaches?	78%	91%
What criteria do you recommend for selecting **mentees** in a programme aimed at female coaches?	60%	90%
What barriers do you think cause **mentors** to lose interest or drop out? What could be done to prevent that?	69%	89%
Which barriers are most likely to cause **mentees** to disengage or drop out of a mentorship programme? What could be done to prevent that?	91%	98%
What strategies can be used to encourage the active involvement of both **mentors** and **mentees** throughout the programme?	93%	96%
What do you think the potential benefits are for female football coaches participating in a mentorship programme?	89%	91%
What do you think the key components of mentorship programme for female coaches should consist of?	91%	97%

## 3 Results

### 3.1 Participants demographics

A three-round expert e-Delphi process was conducted to develop consensus on key components required for a mentorship programme for women football coaches in South Africa. The expert panel initially consisted of thirteen participants (*n* = 13), comprising five females and seven males. Following round 1 of the Delphi process, one male participant withdrew, resulting in a final sample of twelve experts (*n* = 12) (Table 1). The expert panel consisted of individuals with experience in football coaching, coach education, sport administration, and women's football development. Experts represented a range of football contexts, including national and provincial football structures, higher education institutions, community, semi-professional, and professional football environments. Years of professional experience ranged from 5 to 23 years. Data from one participant who completed only the first round were retained to capture wider expert input. As shown in Table 1.

### 3.2 Delphi rounds

#### 3.2.1 Round 1

The e-Delphi questionnaire link was sent to the panel of experts via email on the 9th of June 2025, and information and consent statements were required to be completed before accessing the questions. Participants were requested to complete the questionnaire (consisting of 11 questions) within 2 weeks. On completion, responses were downloaded and exported into a Microsoft Excel sheet for analysis. The data was coded using an iterative process, with a co-coder verifying the codes and ensuring accuracy. Inter-coder agreement of 78% was initially recorded, with fivestatements not reaching the 75% threshold (51). After the discussion between coders, a consensus was reached, achieving an overall agreement level of 92% (Table 2). The formula by Miles and Huberman (52) was used during this phase to ensure scientific rigour and congruency between the researcher and the co-coder. A set of statements was developed based on expert responses to the open-ended questions. Thematic analysis of these responses yielded six overarching themes, representing core mentorship needs (**theme 1:** Mentorship purpose and developmental focus; **theme 2:** Mentor attributes and competencies; **theme 3**: Mentee attributes and responsibilities and functional roles, **theme 4**: Relational and interpersonal dynamics and contextual considerations, **theme 5**: Programme structure, processes, and resources; **theme 6**: Barriers to effective mentorship) within the mentorship framework.

**Table 2:** Demographic information.

**Gender**	**Years of coaching**	**Role**	**Highest coaching qualification**
Female	5	Sport administrator	CAF B
Female	12	Coach	CAF A
Female	10	Instructor	CAF B
Female	15	Instructor	CAF A
Female	11	Coach	CAF B
Male	20+	Mentor	CAF A
Male	13	Instructor	CAF A
Male	10	Coach	CAF A
Male	16	Mentor	CAF A
Male	5	Coach	CAF B
Male	7	Coach	CAF B
Male	8	Coach	CAF B

##### 3.2.1.1 Theme 1: mentorship purpose and developmental focus

Experts described mentorship as a developmental and empowering process aimed at facilitating knowledge transfer, leadership development, and professional growth. Mentorship was viewed as a reciprocal relationship that provides ongoing, tailored support and expert-led guidance. It also fosters empowerment and confidence among women football coaches. Emphasis was placed on structured leadership development, knowledge sharing, and continuous learning as central outcomes of the mentorship process.

##### 3.2.1.2 Theme 2: mentor attributes and competencies

Experts identified a range of attributes considered essential for effective mentors within women's football coaching. These included extensive coaching experience, leadership capability, ethical conduct, effective communication skills, and a supportive coaching approach. Mentors were also expected to demonstrate gender awareness (for example, an understanding of gender-based barriers and inequalities within football, such as unequal hiring practices, biased perceptions of women's competence, and restricted access to professional development opportunities), alongside diversity competence, advocacy for inclusion, and the ability to empower and inspire mentees. Networking capacity, community standing, accessibility, and a history of mentoring were further highlighted as important mentor characteristics.

##### 3.2.1.3 Theme 3: mentee attributes and responsibilities

The role of the mentee was characterised by accountability, commitment, and active engagement in the mentorship process. Experts highlighted the importance of goal setting, openness to feedback, initiative-taking, and reflective practice. Mentees were expected to demonstrate respect for professional boundaries, maintain open communication, adhere to ethical standards, and actively seek guidance to support their personal and professional development. A future-focused mindset and long-term engagement were also emphasised.

##### 3.2.1.4 Theme 4: relational and interpersonal dynamics

Effective mentorship was linked to the quality of the mentor-mentee relationship. Experts emphasised trust-based, supportive relationships characterised by consistent communication, mutual respect, and positive reinforcement. Interpersonal qualities, emotional support, and the mentor's role as a confidant were identified as critical to fostering a safe and enabling environment for learning, growth, and confidence development.

##### 3.2.1.5 Theme 5: programme structure, processes, and resources

Experts highlighted the importance of a clearly defined and well-structured mentorship programme. Key considerations included programme orientation, transparent pairing criteria, shared goal setting, progress tracking, and ongoing evaluation. Adequate tools and resources, access to networking opportunities, financial and organisational support, and alignment with broader coach education pathways were also identified as essential to programme effectiveness and sustainability.

##### 3.2.1.6 Theme 6: barriers to effective mentorship

Experts identified several barriers that could hinder the success of mentorship programmes for women football coaches. These included inconsistent or ineffective communication, time and availability constraints, work-life imbalance, unclear programme structures, and lack of tailored support. Broader contextual challenges such as cultural norms, power imbalances, bias related to gender or race, geographic limitations, and inadequate infrastructure were also noted. Poor programme orientation, insufficient feedback, and pairing mismatches were highlighted as factors that could negatively impact mentorship relationships.

#### 3.2.2 Round 2

During this round, the statements derived from the six themes identified in round 1 were distributed to the expert panel for evaluation. The link was sent to the experts on the 11th of July 2025, and they were asked to complete the questionnaire within 2 weeks. Experts were requested to rate their level of agreement with each statement using a five-point Likert scale ranging from strongly disagree (1) to strongly agree (5) or to suggest changes. The statements generated during this round were grouped into themes to enhance interpretability. While consensus was assessed at the individual statement level, the thematic organisation allowed for a more coherent presentation of the results (Appendix A).

Consensus was defined *a priori* as ≥75% agreement, operationalised as the proportion of experts selecting either *agree* (4) or *strongly agree* (5) for each statement. Statements meeting this criterion were considered to have achieved consensus and were retained for further consideration. Most statements achieved the predefined consensus threshold, indicating strong expert endorsement of the proposed components and principles of a mentorship programme for women football coaches in South Africa. Statements related to the *purpose and dynamics of mentorship*, including developmental support, trust, and sustained engagement, demonstrated particularly strong agreement among experts.

Similarly, high levels of consensus (100%) were reached for statements addressing *the roles, attributes, and responsibilities of mentors and mentees*, with experts strongly endorsing the importance of mentor credibility, leadership experience, ethical conduct, and gender-aware practice. Statements emphasising mentee commitment, goal orientation, reflective practice, and accountability were also widely supported (92%).

Statements related to *programme design, structure, and support mechanisms* achieved substantial consensus (83%) with experts highlighting the necessity of clear programme objectives, structured mentor-mentee matching, goal setting, monitoring and evaluation processes, and alignment with existing coaching pathways. Access to institutional support, networking opportunities, and appropriate resources was also strongly endorsed (83%). Although all items reached consensus in this round, four statements concerning *barriers to mentorship* demonstrated lower levels of agreement (75%), despite remaining above the established threshold.

Two experts disagreed with statements regarding work-life imbalance, lack of recognition, and financial support in Table 3, and some statements received no agreement or comment. It was not determined whether these dissenting views originated from the same individuals, as responses were anonymised. These statements were retained as consensus had been reached among the experts, with the ≥75% level being reached.

**Table 3:** Disagreement comments from experts in round 2.

**Question**	**Statement**	**Comment**
What barriers do you think cause mentors to lose interest or drop out? What could be done to prevent that?	Work-life balance	Work-life balance should not be a determining factor in the quality of work you produce or the effort you put in. Personally, such a role should not have been considered by an individual who knows full well that this role will be highly involved and require x, y, and z.
	Lack of recognition and incentives	Mentorship is expected to happen voluntarily, and many of us already carry heavy coaching and administrative workloads. While recognition and incentives can be helpful, the programme should focus on practical support and structured guidance.
What strategies can be used to encourage the active involvement of both mentors and mentees throughout the programme?	Financial support	Relying on financial support is not realistic or sustainable. Funding in football is inconsistent, and a mentorship programme should be based on professional commitment and structured support rather than financial incentives that may not be available long-term.

#### 3.2.3 Round 3

Round 3 focused on confirming consensus on the mentorship components by presenting experts with full definitions developed from the statements generated and refined in the previous rounds. In addition, a mentorship programme cycle was attached for expert review to provide contextual clarity and support final validation. Experts were asked to indicate whether they would approve or reject the provided statements. Anonymised group feedback from round 2 was provided to inform final evaluations.

Twelve experts (*n* = 12) participated in Round 3. All retained statements met the predefined consensus criterion of greater than 75% agreement and were therefore accepted as final mentorship components for mentorship programme development (Table 4). Despite high overall agreement, experts expressed divergent views regarding the proposed duration of the mentorship programme cycle. In line with Delphi principles, this lack of agreement was not disregarded but used to inform the design of the mentorship framework. Specifically, the final framework will adopt a flexible approach to programme duration, allowing for adaptation at the implementation stage through stakeholder input, rather than enforcing a uniform structure. This flexibility is particularly important in the South African context, where resource availability and institutional capacity may vary. These variations reflected differing perspectives on feasibility, programme intensity, and specific contextual demands. These nuances did not undermine the consensus achieved on the core mentorship components.

**Table 4:** Statements and definitions in round 3.

Sub-theme	Statement	Definition	Consensus (%)
Mentorship definition:		Mentorship is a reciprocal development relationship between an experienced coach and an emerging coach, designed to foster growth through knowledge transfer, expert-led guidance, and structured leadership and coaching. It provides ongoing, tailored support that empowers female coaches to build confidence, enhance their skills, and progress along their coaching pathway. By combining empowerment with practical learning, mentorship creates a collaborative space where both mentor and mentee benefit from shared experiences and insights.	100%
Participants:		CAF A/B female and/or male mentors, SAFA D female mentees.	100%
Roles and responsibilities of a mentor:			
	Knowledge sharing	Share professional expertise, insights, and lived experiences relevant to the mentee's goals	100%
	Tailored support	Offer guidance that is personalised to the mentee's career stage, challenges, and aspirations rather than a “one-size-fits-all” approach.	100%
	Providing constructive feedback	Give feedback on areas such as decision-making, performance in specific tasks (e.g., coaching sessions, leadership responsibilities), or interpersonal skills.	100%
	Ongoing evaluation	Continuously assess the mentee's progress against agreed goals and objectives.	100%
	Confidant	Provide a safe, non-judgmental space for the mentee to discuss challenges, fears, and aspirations.	100%
Roles and responsibilities of a mentee:			
	Learning and being accountable	Be accountable for agreed tasks, deadlines, and responsibilities within the mentorship relationship.	100%
	Remain committed	Dedicate time and energy to the mentorship programme by attending scheduled meetings and actively participating	100%
	Reflect and act on feedback in a constructive manner	Accept constructive feedback with an open mind and use it to improve skills, knowledge, and behaviour	100%
	Goal setting	a) Work collaboratively with the mentor to set realistic short- and long-term goals.	100%
		b) Regularly review progress against these goals and adjust them when necessary.	
	Maintain ongoing and open communication	a) Communicate honestly and regularly with the mentor about progress, challenges, and achievements.	100%
		b) Provide feedback to ensure the relationship remains balanced and beneficial.	
Potential benefits of a mentorship programme:			
	Networking and industry connections	Provides access to valuable contacts and opportunities within the field.	100%
	Knowledge sharing	Allows mentors to transfer experience and insights to mentees	100%
	Personal development	Builds confidence, resilience, and self-awareness	100%
	Skill development	Enhances both technical and soft skills needed for growth	100%
	Career development	Supports progression, goal achievement, and professional advancement.	100%
	Interpersonal qualities	Improves communication, empathy, and relationship-building.	100%
Programme structure:			
	In your opinion, what would be an ideal duration for this mentorship relationship?		50% (6 months)
			8.3% (5 months, 6–12 months, it can be longer, and 12 months or more)
			16.7% (12 months)

**Figure 4:**
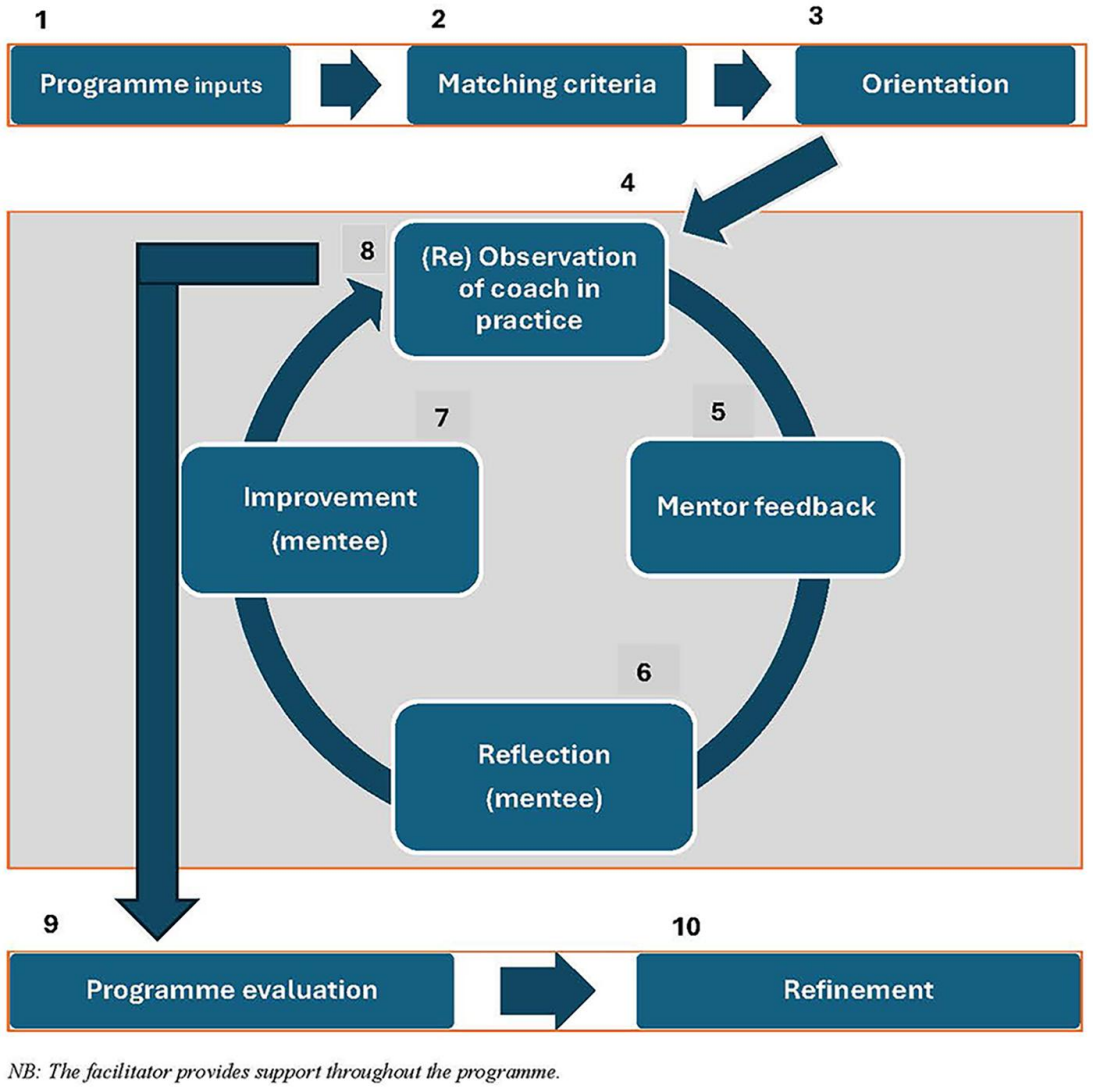
The hybrid mentorship programme for women football coaches in South Africa.

All statements were retained throughout the rounds, including those that reflected minimal or partial disagreement. This decision was made to ensure transparency in reporting the full range of expert perspectives and to avoid the premature exclusion of potentially contextually relevant insights. Rather than being viewed as methodological weakness, limited disagreement was interpreted as meaningful variation in expert opinion, which is particularly important in applied sport coaching contexts where implementation conditions may differ across settings. Therefore, the consensus findings were synthesised into a structured mentorship programme to demonstrate how the Delphi-derived components collectively informed programme development. Figure 4 and Table 5 present the proposed hybrid mentorship programme for women football coaches in South Africa

**Table 5:** Definitions for Figure 4.

**Components**	**Descriptions**
Programme inputs	Institutional support, recruitment of qualified mentors, access to training and development opportunities, stakeholder involvement (e.g., SAFA and football structures), and availability of resources to support mentorship activities.
Matching criteria	Pairing mentors and mentees based on coaching expertise, personality compatibility and experience and professional goals.
	Mentee: SAFA D, emerging coaches, passionate and committed, goal orientated, future-focused mindset and maintains ongoing and open communication.
	Mentor: CAF A/B female and/or male mentor, seasoned and experienced coach, open-mindedness, supportive coaching approach and the ability to empower and inspire.
Orientation and programme preparation	One-page CV containing a brief profile of the mentee and mentor.
	Number of sessions: Predetermined schedule (e.g., monthly/quarterly), agreed upon at the start of the programme.
	Goals & expectations outlining what the mentee hopes to achieve.
	Clarification of mentor and mentee roles and responsibilities (Table 4).
	Signing of a mentorship agreement outlining confidentiality, commitment and communication expectations.
	Use of a logbook or reflective journal to record meetings, progress, and reflections.
Observation of coach in practice	Mentors attend scheduled sessions to observe mentees in practice (mentees' goals will provide greater structure to the sessions).
	Alternate mentor: Another mentor may step in if the main mentor is unavailable.
	Mentor qualities include empowerment, passionate and committed, gender awareness and inclusive leadership and professionalism.
Mentor feedback and support	Mentors provide structured, constructive and ongoing feedback during and after sessions to guide the development and improve coaching practice.
	Mentors should be reliable, acts as confidant, encourage confidence and provides ongoing, tailored support.
	Mentees use the logbook or journal to document meetings, feedback received and reflections.
Reflection and professional development (mentee)	Mentees critically reflect on their coaching experiences, track progress in the journal, seek guidance and clarity where necessary, and acknowledge mentor contributions.
	Reflection should encourage continuous learning, self-awareness, and professional growth.
Improvement (mentee)	Mentees apply strategies and recommendations provided by the mentor during coaching practice and keep notes for discussion in future sessions.
	Mentors monitor the mentee's progress, growth, and application of newly acquired skills while maintaining open communication throughout the programme.
Re-observation of coach in practice and continuous improvement	Following implementation of feedback, mentees return to the field to apply suggested strategies and are re-observed by the mentor to assess improvement and identify further developmental needs.
Programme evaluation and outcomes	Programme outcomes and participant experiences are assessed through feedback, reflective journals, and progress reviews.
	Benefits of participation may include knowledge sharing, networking opportunities, industry connections, personal growth, career advancement, leadership development, and improved coaching confidence.
Refinement	Refinement of the mentorship programme will be undertaken following the programme evaluation to improve its effectiveness, structure, and overall implementation.

## 4 Discussion

The study aimed to establish expert consensus on the key elements required to inform the development of a mentorship programme for women football coaches in South Africa. The findings demonstrate strong consensus regarding the conceptualisation of mentorship, the defining characteristics and competencies of mentors and mentees, perceived barriers, and essential programme components. These results align with and extend existing literature on women's coaching development pathways by providing empirically grounded insights from a Global South football context, where formalised mentorship structures remain underdeveloped.

### 4.1 The concept of mentorship as a structured developmental process

The panel of experts in this study reached consensus that mentorship should be understood as more than an informal or *ad hoc* exchange of advice or support. Instead, mentorship was conceptualised as a structured, ongoing, and reciprocal developmental relationship between the mentor and the mentee. This aligns with mentorship frameworks in sport and organisational literature, which emphasise sustained engagement, reciprocal learning, and goal orientation rather than a once-off or informal interaction (19).

Evidence from a recent systematic review on mentorship programmes for women in coaching and leadership demonstrates that formalised mentorship structures are consistently associated with positive outcomes, including career progression, expanded professional networks, enhanced confidence, and improved retention within coaching pathways (2). Similarly, Banwell, Kerr, and Stirling (20) found that participation in a formal female coach mentorship programme supported development across multiple ecological levels, including individual confidence, interpersonal support networks, and organisational navigation. The review further emphasises that mentorship is most effective when embedded within organisational systems, rather than relying on informal or self-initiated relationships, particularly in contexts characterised by gendered power dynamics and limited institutional support (2).

Within women's football, mentorship has been identified as a critical mechanism for navigating male-dominated coaching environments and supporting long-term career progression (2, 21, 22). Expert consensus in the present study supports this, emphasising that effective mentorship for women coaches must extend beyond technical instruction to address empowerment, confidence, professional identity, and a sense of belonging, factors central to women's sustained participation in coaching (2, 23, 24). This multidimensional focus aligns with Banwell et al.'s (20) ecological analysis, which emphasises that mentorship benefits women coaches not only through skill development but also by strengthening psychosocial support and access within gendered sport systems.

In the South African context, where women football coaches often follow a non-linear entry pathway and experience limited organisational backing, the findings in this study reinforces the need for intentional, formalised, and context-sensitive mentorship programmes. Such programmes are essential for addressing structural inequities and supporting women's long-term development, rather than relying on informal or short-term mentoring arrangements (2).

Due to the dearth of football-specific mentorship research, this discussion draws on broader sporting and organisational. Existing research remains largely concentrated in Global North contexts and is often framed by generic organisational models, with limited sport-, gender-, and context-specific application (2). Consequently, this study contributes by contextualising and supporting established mentorship principles within the South African women's football environment, where structural conditions, resource constraints, and gendered organisational dynamics differ significantly from those represented in existing literature.

### 4.2 Mentee roles and engagement in a mentorship relationship

The findings indicate that experts view mentees as active participants in the mentorship process, with strong consensus regarding responsibilities such as goal setting, commitment, openness to feedback, and ongoing communication. This aligns with the mentorship literature that frames effective mentoring as a reciprocal developmental relationship rather than a passive transfer of knowledge (25–28). Experts further agreed that mentee selection should prioritise motivation, commitment, and engagement at the beginning stages of the relationship, showing that mentorship is effective when mentees demonstrate readiness for development and learning (29). Notably, the experts identified potential barriers to mentee engagement, including unclear expectations, inconsistent communication, power imbalances, lack of trust, and competing work-life constraints. In line with research regarding women's coaching experiences, these barriers highlight the need for a structured, supportive mentorship programme cycle that fosters trust, clarity, and long-term participation and engagement for women coaches in South Africa (30). This aligns with Banwell et al. (20), who demonstrated that formal mentorship programmes support women coaches' developmental engagement not only through goal attainment, but also through enhanced confidence, identity formation, and access to professional support structures. This supports existing evidence that structured mentorship programmes with clear expectations and regular evaluation are more likely to retain mentees and facilitate meaningful development outcomes (19, 53).

### 4.3 Mentor credibility, experience, and competence

Experts strongly agree that mentors should possess coaching competence, relevant experience, passion, and commitment, reinforcing the importance of credibility in mentoring relationships. This finding reflects existing research indicating that mentors who possess contextual understanding and have a proven track record or recognised expertise are more likely to be perceived as legitimate and influential, particularly in male-dominated environments (31, 32).

While networking and industry connections were broadly supported, the greater variability in responses suggests that experts regarded these elements as beneficial but insufficient in isolation. This pattern may reflect an awareness that access to networks within South African football is uneven and frequently shaped by entrenched structural inequalities (33, 34). As such, mentorship programmes should not only leverage existing networks but also actively create new pathways and opportunities for women coaches who have historically been excluded from the football environment.

### 4.4 Organisational and structural barriers to mentorship

A key contribution of this study is the establishment of expert consensus on systemic barriers to mentorship, including time and availability constraints, excessive workloads, and the absence of formal recognition or incentive structures for mentors. These findings align with existing research indicating that women coaches are frequently expected to undertake additional emotional and developmental labour within sport systems, often without recognition, institutional support, or material reward (24, 35–37).

In South Africa, where mentorship is frequently assumed to occur informally and voluntarily, experts' responses highlight the unsustainability of such expectations. Similar concerns have been raised in international literature, where the absence of institutional support has been shown to limit the effectiveness and longevity of mentorship programmes (8, 38–41). The findings reinforce the need for mentorship to be structurally embedded within coach education, development pathways, and football association systems, rather than being positioned as an optional, peripheral, or informal activity. This is consistent with Banwell et al.'s (20) ecological analysis of a female coach mentorship programme, which highlights that sustained developmental impact is dependent on organisational recognition, structural support, and alignment with broader coaching systems. Experts noted that mentorship effectiveness may be undermined by mismatches in mentor-mentee pairings, unclear role expectations, inconsistent communication, and misalignment of goals or coaching philosophies. This suggests that even when mentorship structures exist, poorly managed implementation can undermine relationship quality and developmental outcomes.

The experts' views are reflected in the broader mentorship literature, which cautions that mentoring relationships are highly context-dependent and vulnerable to breakdown or failure when pairing processes and expectations are not clearly defined (19, 42–44). The identification of these challenges highlights the importance of structured pairing criteria, clear orientation processes, and ongoing programme oversight to support effective mentor-mentee relationships, particularly in complex and resource-constrained football environments.

### 4.5 Key components of an effective mentorship programme

Experts reached strong agreement on the inclusion of structured components such as clear mentor-mentee pairing, programme orientation, experiential learning, skill development, and regular feedback. Empirical evidence from a formal female coach mentorship programme further supports this approach, demonstrating benefits across individual, interpersonal, and organisational levels when programmes are intentionally designed and supported (20). These elements reflect best-practice mentorship models identified in sport and leadership research, which emphasise clarity of roles, structured engagement, and ongoing evaluation (2, 45, 46).

Experts strongly supported advocacy, equity, and networking components, highlighting the gendered undertone of coaching development. This aligns with research arguing that mentorship programmes for women must explicitly address structural inequality and not assume gender-neutral environments (47, 54, 55). In football specifically, Culvin and Bowes (53) note that mentorship initiatives are most effective when they actively challenge exclusionary practices and create alternative pathways to leadership.

The emphasis on experiential learning is further reflected in literature highlighting the importance of access to high-quality coaching environments, observation opportunities, and applied learning areas where women coaches have historically been marginalised (23, 24).

## 5 Conclusion

This study aimed to establish expert consensus on the key components required to inform the design of a structured mentorship programme for women football coaches in South Africa. Using an e-Delphi approach, the study addressed the lack of empirical guidance for mentorship design within a historically male-dominated and resource-constrained football context.

Synthesising expert perspectives across coaching, coach education, and football administration, the findings highlight that effective mentorship for women football coaches must be intentionally structured, embedded within formal coaching pathways, and supported at organisational level. Essential components include transparent mentor–mentee matching, clearly defined roles and expectations, capacity-building opportunities, relational support, and ongoing monitoring and evaluation. Importantly, mentorship was consistently viewed not as an informal or voluntary relationship, but as a developmental mechanism requiring institutional recognition and alignment with broader coach development systems.

The key contribution of this study lies in the development of a context-specific mentorship framework that has the potential to transform women's football coaching pathways by expanding access to developmental support, strengthening professional networks, and supporting sustained career progression in South Africa. By embedding mentorship within formal football structures, this framework offers a practical strategy to move beyond *ad hoc* support and toward more equitable, sustainable, and intentionally designed coaching development systems for women in South African football. In conclusion, this study presents a consensus-based mentorship framework for women football coaches and is, to the best of the authors' knowledge, the first of its kind in the South African context.

## 6 Implications

The findings of this Delphi study have several implications for policy and practice in the development of mentorship programmes for women football coaches. At a policy level, the agreed-upon components of the framework can inform the development of structured support systems aimed at advancing women's participation and progression within coaching pathways.

In practice, the final Delphi-derived framework provides a structured yet adaptable guide for implementing mentorship programmes within sport organisations, allowing for contextual adjustments based on available resources and institutional capacity. This reflects both areas of consensus and the retained variation in expert perspectives across rounds.

In terms of future implementation, the flexible nature of the framework supports its adaptation across different sport environments and levels of coaching development. This adaptability is particularly important in contexts where organisational structures and resource availability vary, such as in South African sport systems. Future application of the framework may therefore involve iterative refinement through stakeholder engagement to ensure sustained relevance, usability, and effectiveness.

## 7 Limitations and future studies

These are some of the limitations that could be woven into this section. They deal mainly with methodological limitations of the e-Delphi method, sampling, and scope:

1. The use of a consensus-based e-Delphi design prioritises expert agreement, which may suppress minority or dissenting perspectives that could be theoretically or practically valuable.
2. Reliance on expert opinion means findings reflect perceptions and professional experiences rather than direct empirical observation of mentorship practices or outcomes.
3. Potential selection bias exists, as participation depended on availability, willingness, and access to digital platforms.
4. Future studies using Delphi methodology in this context are encouraged to apply more robust reliability measures, such as Cohen's kappa or Krippendorff's alpha to strengthen the assessment of coder agreement.

Future research should focus on developing and piloting the mentorship framework within women's football structures, including longitudinal evaluation of its impact on coach retention and career progression. Further studies exploring the lived experiences of mentors and mentees, as well as comparative research across sporting codes or contexts, would strengthen understanding of how structured mentorship can be adapted and scaled within women's sport. The findings are context-specific to South African football and may not be directly generalisable to other sporting codes or national contexts without adaptation. SAFA may also have its own structural, cultural and institutional dynamics that may limit transferability to other contexts.

## Data Availability

The original contributions presented in the study are included in the article/Supplementary Material, further inquiries can be directed to the corresponding author.
