# Supplementary material for: Designing a mentorship programme for women football coaches in South Africa: an expert e-Delphi study
Source: Front Sports Act Living. 2026 Jul 6;8:1821565. doi: 10.3389/fspor.2026.1821565 (PMC13381308; doi:10.3389/fspor.2026.1821565)
Supplement: Supplementary file 1 [file Datasheet1.pdf]

List of statements

Question 1: How would you define the term mentorship?

Statement	Level of agreement
Knowledge Transfer	100%
Ongoing, tailored support	100%
Expert-led guidance	100%
Structured leadership and coaching	92%
Empowerment	100%
Reciprocal development relationship	92%

Overall (%) = 97

Question 2: What qualities and experiences should **mentors** possess to effectively support and guide female mentees in the football industry?

Statement	Level of agreement
Coaching competence and experience	100%
Networking and industry connections	83%
Passion and commitment	100%
Interpersonal qualities	100%
Gender awareness	100%
Advocacy and inclusion	100%
Inclusive Leadership	100%
Diversity competence	100%
Empowerment	100%

Overall (%) = 98

Question 3: What do you think the roles and responsibilities are for a **mentor** in a mentorship programme?

Statement	Level of agreement
Knowledge sharing	100%
Ongoing, tailored support	100%
Provide feedback for growth	100%
Advocacy and inclusion	92%
Maintaining professionalism	100%
Ongoing evaluation	92%
Empowerment	100%
Confidant	100%

Overall (%) = 98

Question 4: What do you think the roles and responsibilities are for a **mentee** in a mentorship programme?

Statement	Level of agreement
Learning and being accountable	100%
Remain committed	100%
Reflect and act on feedback in a constructive manner	100%
Goal setting	100%
Seek guidance and clarity	100%
Take initiative	100%
Maintain respect and boundaries	100%
Maintain ongoing and open communication	100%
Acknowledge mentor's contributions	92%
Abide by ethical principles and professional standards	100%
Learning and being accountable	100%

Overall (%) = 99

Question 5: What criteria do you recommend for selecting **mentors** in a programme aimed at female coaches?

**Statement**	**Level of agreement**
Seasoned and experienced coach	83%
Open mindedness	100%
Supportive coaching approach	100%
Leadership	100%
Community standing and reputation	75%
Diversity competence	92%
Interpersonal qualities	100%
Networking and industry connection	83%
Advocacy and inclusion	100%
Ability to empower and inspire	100%
Effective communication	100%
Coach curiosity/receptibility	100%
Leadership capability	100%
Reliable or accessible	100%
Ethical conduct	100%
Mentoring history	75%

Overall (%) = 94

Question 6: What criteria do you recommend for selecting **mentees** in a programme aimed at female coaches?

**Statement**	**Level of agreement**
Emerging coaches	75%
Passion and commitment	100%
Coach curiosity/receptibility	92%
Long term engagement	83%
Educational foundation	75%
Future-focused mindset	100%
Goal-oriented	100%
Expert-led guidance	92%
Diversity competence	75%
Interpersonal qualities	100%
Grassroot and community involvement	92%
Leadership development	100%

Overall (%) = 90

Question 7: What barriers do you think cause **mentors** to lose interest or drop out? What could be done to prevent that?

Statement	Level of agreement
Inconsistent communication	92%
Lack of trust	83%
Minimal engagement	92%
Time and availability constraints	92%
Ineffective communication	92%
Poor commitment and disengagement	100%
Work-life imbalance	92%
Unclear programme structure	92%
Lack of recognition or incentives	67%
Pairing mismatch	58%
Lack of tailored support	83%
Cultural norms and traditions	67%
Limited access due to location and poor infrastructure	92%
Bias against race or gender	67%
Poor programme orientation	92%
Insufficient feedback	83%
Undefined goals and expectations	83%

Overall (%) = 84

Question 8: Which barriers are most likely to cause **mentees** to disengage or drop out of a mentorship programme? What could be done to prevent that?

Statement	Level of agreement
Inconsistent communication	92%
Lack of trust	100%
Power dynamics or imbalance	100%
Insufficient support	100%
Interpersonal challenges	100%
Pairing mismatch	83%
Time and availability constraints	100%
Undefined goals and expectations	92%
Lack of tailored support	100%
Time and availability constraints	100%
Minimal engagement	92%
Lack of developmental opportunities (personal or professional)	83%
Insufficient feedback	100%
Unclear programme structure	100%
Work-life imbalance	92%

Overall (%) = 96

Question 9: What strategies can be used to encourage the active involvement of both **mentors** and **mentees** throughout the programme?

Statement	Level of agreement
Consistent communication	100%
Accountability	100%
Collaborative learning	100%
Trust based relationship	100%
Evaluation and improvement for growth	100%
Interpersonal qualities	100%
Programme planning, design and/or structure	92%
Progress tracking and setting of shared goals	100%
Programme orientation	100%
Pairing process and criteria	92%
Positive reinforcement	100%
Financial Support	83%
Team management and support	92%
Tools & Resources	92%

Overall (%) = 97

Question 10: What do you think the potential benefits are for female football coaches participating in a mentorship programme?

Statement	Level of agreement
Knowledge sharing	100%
Networking and industry connections	92%
Personal development	100%
Team management and support	100%
Skill development	100%
Interpersonal qualities	92%
Career development	100%
Advocacy and inclusion	100%
Leadership development	100%

Overall (%) = 98

Question 11: What do you think the key components of mentorship programme for female coaches should consist of?

Statement	Level of agreement
Supportive relationship and environment	100%
Emotional wellbeing	83%
Team management and support	100%
Coach curiosity/receptibility	100%
Programme planning, design and/or structure	100%
Evaluation and improvement for growth	100%
Pairing process or and criteria	75%
Skill development	100%
Advocacy for women in coaching	92%
Programme orientation	100%
Interpersonal qualities	100%
Networking and industry connection	83%
Experiential learning	100%
Equity and inclusion	100%
Positive reinforcement	100%

Overall (%) = 96
